# Angiotensin II receptor 1 gene variants are associated with high-altitude pulmonary edema risk

**DOI:** 10.18632/oncotarget.12489

**Published:** 2016-10-06

**Authors:** Tianbo Jin, Yongchao Ren, Xikai Zhu, Xun Li, Yongri Ouyang, Xue He, Zhiying Zhang, Yuan Zhang, Longli Kang, Dongya Yuan

**Affiliations:** ^1^ Key Laboratory for Molecular Genetic Mechanisms and Intervention Research on High Altitude disease of Tibet Autonomous Region, School of Medicine, Xizang Minzu University, Xianyang, Shaanxi 712082, China; ^2^ Key Laboratory of High Altitude Environment and Genes Related to Diseases of Tibet Autonomous Region, School of Medicine, Xizang Minzu University, Xianyang 712082, China; ^3^ Key Laboratory for Basic Life Science Research of Tibet Autonomous Region, School of Medicine, Xizang Minzu University, Xianyang, Shaanxi 712082, China; ^4^ School of Life Sciences, Northwest University, Xi'an, Shaanxi 710069, China; ^5^ Qiannan Institute for Food and Drug Control, Duyun, Guizhou 558000, China; ^6^ The Center of Altitude Disease, General Hospital of Tibet Military Area Command, Lasa 850000, China

**Keywords:** high-altitude pulmonary edema (HAPE), AGTR1, polymorphisms, haplotype

## Abstract

Previous studies demonstrated that Angiotensin II Receptor 1 (*AGTR1*) may play an important role in the development of high-altitude pulmonary edema. We envisaged a role for *AGTR1* gene variants in the pathogenesis of HAPE and investigated their potential associations with HAPE in a Han Chinese population. We genotyped seven *AGTR1* polymorphisms in 267 patients with diagnosed HAPE and 304 controls and evaluated their association with risk of HAPE. Statistically significant associations were found for the single nucleotide polymorphisms (SNPs) rs275651 (*p* = 0.017; odds ratio [OR] = 0.65) and rs275652 (*p* = 0.016; OR = 0.64). Another SNP rs10941679 showed a marginally significant association after adjusting for age and sex in the additive genetic model (adjusted OR = 1.44, 95% CI = 1.01-2.04, *p* = 0.040). Haplotype analysis confirmed that the haplotype “AG” was associated with a 35% reduction in the risk of developing HAPE, while the haplotype “AA” increased the risk of developing HAPE by 44%. These results provide the first evidence linking genetic variations in *AGTR1* with HAPE risk in Han Chinese individuals.

## INTRODUCTION

High-altitude pulmonary edema (HAPE), a lethal, non-cardiogenic form of pulmonary edema, is a severe form of acute mountain sickness occurring in susceptible individuals at altitudes >2,500 m above sea level. It is an acute idiopathic disease that presents within 2 to 4 days after arrival at high altitude [1, 2] and can occur in two forms. The first form typically occurs in rapidly ascending un-acclimatized healthy lowlanders. The second form, also called re-entry HAPE, occurs in high-altitude dwellers who return from a sojourn at a low altitude. The two forms probably have the same pathophysiological basis [3]. The incidence of HAPE is not definitively known, since it commonly occurs in remote mountainous regions, where data are often unavailable, and some patients may recover fully during descent [4]. Early symptoms of HAPE include cough, exertional dyspnea, and reduced exercise performance. As HAPE progresses, cough worsens and breathlessness at rest and sometimes orthopnea occur [3, 5]. It is generally known that high-altitude pulmonary edema tends to be associated with exaggerated pulmonary hypertension.

Since the response to the extreme high altitude environment varies among individuals and those who have previously experienced HAPE run a significant risk of recurrence, it has been speculated that a constitutional or genetic component may underlie the development of this disease [6]. To date, a number of candidate genes, such as angiotensin-converting enzyme (*ACE*), angiotensin (*AGT*), renin (*REN*), aldosterone synthase (*CYP11B2*), and angiotensin II receptor type 1 (*AGTR1*), have been reported to be involved in the physiological response to hypoxic conditions. These genes have been associated with high risk of susceptibility to HAPE in some populations [7, 8].

As whole genome-wide association studies have not identified common variants in or near the *AGTR1* gene associated with HAPE, the current study evaluated 7 SNPs that span the *AGTR1* gene and their associations with HAPE risk in a case-control model comprising 571 individuals from a Han Chinese population.

## RESULTS

A total of 267 patients with HAPE (aged 32.57 ± 10.738 years) and 304 healthy controls (aged 36.15 ± 4.498 years) were included in the current study. Principle demographic characteristics of all participants are listed in Table 1 including age and sex. Table 2 lists the detailed information of candidate SNPs tested in the *AGTR1* gene. We examined Hardy–Weinberg equilibrium in the controls, and all SNPs were found to be in Hardy-Weinberg equilibrium (*p* ≥ 0.05) except for the SNP rs2638360 (*p =* 0.042). When comparing allelic frequency of HAPE cases and controls, we found two significant SNPs in the *AGTR1* gene at a 5% level (rs275651, *p* = 0.017, odds ratio [OR] = 0.65, 95% confidence interval [CI] = 0.45-0.93; rs275652, *p* = 0.016, odds ratio [OR] = 0.64, 95% confidence interval [CI] = 0.45-0.92).

**Table 1:** Main demographic characteristics of HAPE cases and controls

	Cases	%	Control	%	*p*^a^
Total	267		304		
Mean ± SD					
Age	32.57 ± 10.738		36.15 ± 4.498		0.000*
Sex					0.105^b^
Female	21	7.9	14	4.6	
Male	246	92.1	290	95.4	

* *p* ≤ 0.05 indicates statistical significance.

a Independent samples t test.

b Two-sided χ2 tests.

**Table 2:** Candidate SNPs tested in the study

SNP ID	Genes	Band	Role	Alleles A/B	*p*-HWE	*p*^a^	OR(95% CI)
rs275651	AGTR1	3q24	Promoter	A/T	0.647	0.017*	0.65(0.45-0.93)
rs275652	AGTR1	3q24	Promoter	G/T	0.647	0.016*	0.64(0.45-0.92)
rs2638360	AGTR1	3q24	Intron	G/A	0.042	0.178	0.75(0.49-1.14)
rs4681443	AGTR1	3q24	Intron	A/G	0.216	0.950	0.99(0.63-1.54)
rs1492099	AGTR1	3q24	Intron	G/A	0.723	0.931	0.99(0.74-1.32)
rs1492097	AGTR1	3q24	Intron	A/G	1.000	0.118	1.31(0.93-1.85)
rs4524238	AGTR1	3q24	Intron	A/G	1.000	0.100	1.33(0.95-1.88)

SNP, single-nucleotide polymorphism; OR, odds ratio; 95%CI, 95% confidence interval; HWE, Hardy–Weinberg equilibrium.

a Two-sided χ2 tests/Fisher's exact tests.

* *p* ≤ 0.05 indicates statistical significance.

The association analysis between the three SNPs (rs275651, rs275652 and rs4524238) and HAPE risk are summarized in Tables 3–5. Our results showed that two SNPs were significantly associated with susceptibility to HAPE in crude analysis by the heterozygote comparison (rs275651 AT vs. TT, OR = 0.55, 95% CI = 0.36-0.83; rs275652 GT vs. TT, OR = 0.55, 95%CI = 0.36-0.83), dominant model analyses (rs275651, OR = 0.58, 95% CI = 0.39-0.86, *p* = 0.007; rs275652, OR = 0.57, 95% CI = 0.38-0.86, *p* = 0.006), and additive model analyses (rs275651, OR = 0.65, 95% CI = 0.45-0.93, *p* = 0.017; rs275652, OR = 0.65, 95% CI = 0.45-0.93, *p* = 0.016). After adjustment for age and sex, these associations remained significant (*p* < 0.05). We also observed another susceptibility SNP, rs4524238, by additive model analyses (adjusted OR = 1.44, 95% CI = 1.01-2.04, *p* = 0.040) after adjusting for age and sex.

**Table 3:** Analysis of association between rs275651 polymorphism and risk of HAPE

	Case (%)	Control (%)	*p*^a^	Crude		Adjusted^b^	
	N = 266	N = 304		OR (95%CI)	*p*	OR (95%CI)	*p*
Genotype							
T/T	218 (82.0%)	220 (72.4%)	0.017*	1	0.016*	1.00	0.018*
A/T	43 (16.2%)	79 (26.0%)		0.55 (0.36-0.83)		0.55 (0.36-0.84)	
A/A	5 (1.8%)	5 (1.6%)		1.01 (0.29-3.54)		1.05 (0.29-3.82)	
Dominant							
T/T	218 (81.9%)	220 (72.4%)	0.007*	1	0.007*	1	0.008*
A/T-A/A	48 (18.1%)	84 (27.6%)		0.58 (0.39-0.86)		0.57 (0.38-0.87)	
Recessive							
T/T-A/T	261 (98.1%)	299 (98.4%)	0.915	1	0.830	1	0.780
A/A	5 (1.9%)	5 (1.6%)		1.15 (0.33-4.00)		1.20 (0.33-4.33)	
Additive							
T/T	218 (82%)	220 (72.4%)	0.018*	0.65 (0.45-0.93)	0.017*	0.65 (0.45-0.94)	0.021*
A/T	43 (16.2%)	79 (26.0%)					
A/A	5 (1.8%)	5 (1.6%)					

a Two-sides χ2 test/Fisher's exact tests.

b Adjusted for age and sex in a logistic regression model.

* *p* ≤ 0.05 indicates statistical significance.

**Table 4:** Analysis of association between rs275652 polymorphism and risk of HAPE

	Case (%)	Control (%)	*p*^a^	Crude		Adjusted^b^	
	N = 267	N = 304		OR (95%CI)	*p*	OR (95%CI)	*p*
Genotype							
T/T	219 (82.0%)	220 (72.4%)	0.016*	1	0.015*	1.00	0.017*
G/T	43 (16.1%)	79 (26.0%)		0.55 (0.36-0.83)		0.54 (0.35-0.83)	
G/G	5 (1.9%)	5 (1.6%)		1.00 (0.29-3.52)		1.05 (0.29-3.81)	
Dominant							
T/T	219 (82.0%)	220 (72.4%)	0.006*	1	0.006*	1	0.007*
G/T-G/G	48 (18.0%)	84 (27.6%)		0.57 (0.38-0.86)		0.57 (0.38-0.86)	
Recessive							
T/T-G/T	262 (98.1%)	299 (98.4%)	0.910	1	0.840	1	0.780
G/G	5 (1.9%)	5 (1.6%)		1.14 (0.33-3.99)		1.20 (0.33-4.32)	
Additive							
T/T	219 (82.0%)	220 (72.4%)	0.017*	0.65 (0.45-0.93)	0.016*	0.65 (0.45-0.94)	0.020*
G/T	43 (16.1%)	79 (26.0%)					
G/G	5 (1.9%)	5 (1.6%)					

a Two-sides χ2 test/Fisher's exact tests.

b Adjusted for age and sex in a logistic regression model.

* p ≤ 0.05 indicates statistical significance.

**Table 5:** Analysis of association between rs4524238 polymorphism and risk of HAPE

	Case (%)	Control (%)	*p*^a^	Crude		Adjusted^b^	
	N = 267	N = 304		OR (95%CI)	*p*	OR (95%CI)	*p*
Genotype							
G/G	196 (73.4%)	237 (78.0%)	0.180	1.00	0.180	1.00	0.082
A/G	62 (23.2%)	63 (20.7%)		1.19 (0.80-1.77)		1.30 (0.86-1.97)	
A/A	9 (3.4%)	4 (1.3%)		2.72 (0.83-8.97)		3.20 (0.94-10.90)	
Dominant							
G/G	196 (73.4%)	237 (78.0%)	0.205	1	0.210	1	0.088
A/G-A/A	71 (26.6%)	67 (22.0%)		1.28 (0.87-1.88)		1.41 (0.95-2.10)	
Recessive							
G/G-A/G	258 (96.6%)	300 (98.7%)	0.100	1	0.098	1	0.065
A/A	9 (3.4%)	4 (1.3%)		2.62 (0.80-8.60)		3.01 (0.89-10.18)	
Additive							
G/G	196 (73.4%)	237 (78.0%)	0.108	1.32 (0.94-1.84)	0.110	1.44 (1.01-2.04)	0.040*
A/G	62 (23.2%)	63 (20.7%)					
A/A	9 (3.4%)	4 (1.3%)					

a Two-sides χ2 test/Fisher's exact tests.

b Adjusted for age and sex in a logistic regression model.

* *p* ≤ 0.05 indicates statistical significance.

**Figure 1:**
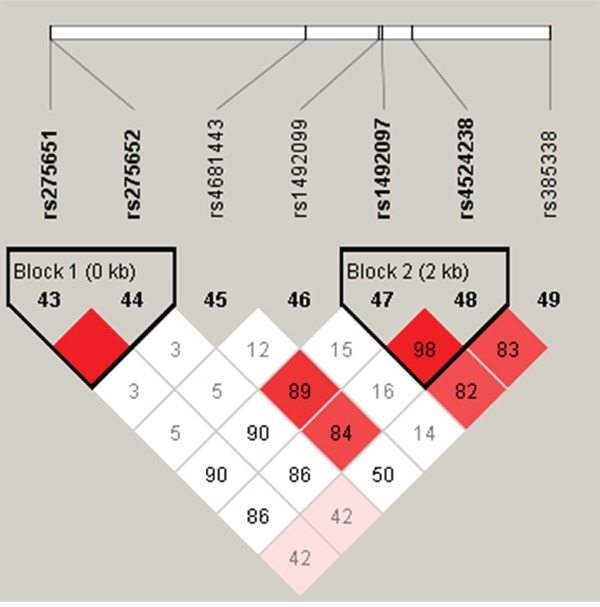
Haplotype block map for all the SNPs of the *AGTR1* gene

Two haplotype blocks were detected when using the D′ measure of linkage disequilibrium (Figure 1). One haplotype block spanned rs275651and rs275652 in promoter region (Haploblock1) and another haplotype block contained rs1492097 and rs4524238 in intron region (Haploblock2). The relationship between *AGTR1* haplotype and the risk of the occurrence of HAPE were listed in Table 6. Haplotype “AG” in Block 1 has been shown to be significantly associated with the risk of HAPE in crude analysis (OR = 0.65, 95% CI = 0.45-0.93, *p* = 0.018), and the result remained significant after adjustment for age and sex (*p* < 0.05). In Block 2, a significant increase risk between the haplotype “AA” and susceptibility of HAPE was only observed after adjustment for age and sex (adjusted OR = 1.44, 95% CI = 1.02 - 2.05, *p* = 0.040).

**Table 6:** *AGTR1* haplotype frequencies and the association with HAPE risk in case and control subjects

Block	Haplotype	Freq (case)	Freq (control)	*p*^a^	Crude		Adjusted^b^	
					OR (95%CI)	*p*	OR (95%CI)	*p*
1	TT	0.901	0.854	0.016*	1		1	
	AG	0.099	0.146	0.016*	0.65 (0.45 - 0.93)	0.018*	0.65 (0.45 - 0.94)	0.022*
2	GG	0.850	0.881	0.124	1		1	
	AA	0.150	0.116	0.087	1.32 (0.94 - 1.85)	0.110	1.44 (1.02 - 2.05)	0.040*

a Two-sides χ2 test/Fisher's exact tests.

b Adjusted for age and sex in a logistic regression model.

* *p* ≤ 0.05 indicates statistical significance.

## DISCUSSION

In this case-control study, we examined the association between polymorphisms in the *AGTR1* gene and propensity to develop HAPE. Two susceptibility loci (rs275651 and rs275652) were associated with decreased risk of HAPE (rs275651, OR = 0.65; rs275652, OR = 0.64), whereas rs4524238 was associated with significantly increased risk (OR = 1.44). Additionally, haplotype-based tests of association indicated a risk effect of up to 44% for “AA” haplotype and a protective effect of up to 35% for “AG” haplotype. To our knowledge, this is the first study to report the effect of these variants in HAPE individuals.

The *AGTR1* gene, which maps to chromosome 3q21–q25 and consists of five exons and four introns spanning approximately 45.1 kb of genomic DNA, encodes the type 1 angiotensin II receptor (AT_1_R). AT_1_R is a 7 transmembrane domain G-protein coupled receptor, widely expressed in different tissues and known to mediate most of the classical actions of Angiotensin II, including vasoconstriction and cellular growth and proliferation [9, 10] [11]. In hypoxic conditions Angiotensin II interacts with AT_1_R to modulate the pulmonary vasoconstrictive response [12]. We speculate that the AGTR1 gene is critical in HAPE pathogenesis its association with HAPE has also been demonstrated in a Japanese population [8]. *AGTR1* gene polymorphisms are frequently observed [13], and a number of different polymorphisms in the *AGTR1* gene have been identified [14]. However, variants of the angiotensin receptor AGTR1 were not found to associate with AMS and HAPE in various ethnic groups in earlier studies [15, 16]. In the current study, seven SNPs (rs275651, rs275652, rs2638360, rs4681443, rs1492099, rs1492097, rs4524238) were genotyped; of these rs275651, rs275652 and rs4524238 were related to HAPE susceptibility. Carriers of the rs275651 “A” allele exhibited a statistically significant decrease of 43% and 35% in HAPE susceptibility by the dominant model and additive model, respectively. rs275652 also decreased risk by 43% and 35%. In haplotype analysis, rs275651 and rs275652 showed a strong linkage disequilibrium pattern and formed one haplotype block. These results indicate that these two variants conferred a possible protective effect against HAPE. Additionally, an additive effect of the rs4524238 “A” allele was observed, resulting in a multiple increased 1.44-fold risk of HAPE. This study is the first to report the rs4524238 variant in any ethnic group and our findings suggest that this variant significantly contributes to the development of HAPE. As the rs4524238 polymorphism is an intronic mutant, the mechanism by which it confers risk is unclear. It is possible that the variant affects the transcription of *AGTR1* or is in linkage disequilibrium with true functional variations directly involved in HAPE susceptibility.

Several potential limitations should be taken into consideration. First, participants in this study were limited to the Han Chinese population; whether the results are also applicable to other ethnic groups is currently unknown. Second, the limited sample size of our study did not allow subgroup analysis. Third, when multiple comparisons were carried out, we did not apply Bonferroni correction to control potential type I errors because this correction method might have increased the chance of a type II error. Finally, additional studies including fine mapping and functional validation are needed to clarify these findings and the mechanisms by which this polymorphism increases HAPE risk.

To sum up, we identified the first significant associations between SNPs rs275651, rs275652 and rs4524238 and HAPE susceptibility, which may provide important biological markers for early diagnosis and prognosis for HAPE patients.

## MATERIALS AND METHODS

### Study participants

From March 2013 to October 2015, we consecutively recruited 267 HAPE patients (HAPE-susceptible cohorts) and 304 ethnically matched control subjects (HAPE-resistant cohorts) from the General Hospital of Tibet Military Region, Lhasa, China. All of the subjects were of genetically unrelated Chinese Han ancestry and informed consent was obtained from each individual enrolled. The patient was immediately admitted to the hospital if anoxic symptoms arose at high altitude. All HAPE patients were diagnosed and confirmed to suffer from HAPE following previously described criteria [17]. A standardized questionnaire and clinical examination were used to exclude any previous history of cardiopulmonary disease. Patients had not received any drug treatment before blood samples were taken. They were all in good health after timely treatment. The control subjects were defined as HAPE- resistant cohorts due to their resistance to HAPE during exposure to similar high-altitude environment. No subjects reported any history of HAPE or other cardiopulmonary disorders in a questionnaire sheet that contained the components of Lake Louise Score [18]. Blood samples were taken from the study participants, and immediately frozen and stored at −70 C until analysis. The study was approved by the Human Research Committee for Approval of Research Involving Human Subjects.

### SNP selection and genotyping

SNPs within the *AGTR1* gene with minor allele frequency (MAF) > 0.05 in the HapMap Asian population were obtained from PubMed databases and single-nucleotide polymorphism (SNP) databases [the dbSNP (NCBI) and the Japanese SNP database (JSNP)]. DNA was extracted from peripheral whole blood cells by the GoldMag-Mini Whole Blood Genomic DNA Purification Kit (GoldMag Co. Ltd. Xian, China) following the manufacturer's protocols, and DNA concentration of each sample was determined by spectrometry (DU530 UV/VIS spectrophotometer, Beckman Instruments, Fullerton, CA, USA). The PCR and extension primers were designed using the MassARRAY Assay Design 3.0 software (Sequenom, Inc.). SNPs were genotyped with the Sequenom MassARRAY RS1000 according to the instructions of the manufacturer [19]. We performed data management and analyses using Sequenom MassArray TYPER 4.0 software.

### Statistical analysis

Statistical analysis was performed using Microsoft Excel and SPSS 16.0(SPSS, Chicago, IL, USA). All hypothesis testing was two-sided with a p value of 0.05 deemed as significant. Observed genotype frequencies for *AGTR1* polymorphisms in controls were tested for deviation from Hardy–Weinberg equilibrium (HWE) using a goodness-of-fit χ2 test. Odds ratios (ORs) and 95% confidence intervals (CI) were estimated by unconditional logistic regression after adjustment for age and sex. The genotypic associations of every polymorphism were compared under the dominant, recessive, and additive genetic models as suggested by previous studies using the same method [20, 21]. Finally, the Haploview software package (http://www.broadinstitute.org/haploview) and the SHEsis software platform (http://analysis.bio-x.cn/myAnalysis.php) was used to analyze the linkage disequilibrium (LD) structure, calculate D′ to define haplotype blocks and estimate haplotype frequencies [22, 23].
